# Brain Gene Expression of a Sporadic (icv-STZ Mouse) and a Familial Mouse Model (3xTg-AD Mouse) of Alzheimer’s Disease

**DOI:** 10.1371/journal.pone.0051432

**Published:** 2012-12-07

**Authors:** Yanxing Chen, Zhu Tian, Zhihou Liang, Shenggang Sun, Chun-ling Dai, Moon H. Lee, Frank M. LaFerla, Inge Grundke-Iqbal, Khalid Iqbal, Fei Liu, Cheng-Xin Gong

**Affiliations:** 1 Department of Neurochemistry, New York State Institute for Basic Research in Developmental Disabilities, Staten Island, New York, United States of America; 2 Department of Neurology, Union Hospital, Tongji Medical College, Huazhong University of Science & Technology, Wuhan, Hubei, China; 3 Department of Neurology, The First Hospital of Jilin University, Changchun, Jilin, China; 4 Department of Developmental Neurobiology, New York State Institute for Basic Research in Developmental Disabilities, Staten Island, New York, United States of America; 5 Department of Neurobiology and Behavior, University of California Irvine, Irvine, California, United States of America; Centre Hospitalier de l'Université Laval, Canada

## Abstract

Alzheimer’s disease (AD) can be divided into sporadic AD (SAD) and familial AD (FAD). Most AD cases are sporadic and may result from multiple etiologic factors, including environmental, genetic and metabolic factors, whereas FAD is caused by mutations of presenilins or amyloid-β (Aβ) precursor protein (APP). A commonly used mouse model for AD is 3xTg-AD mouse, which is generated by over-expression of mutated presenilin 1, APP and tau in the brain and thus represents a mouse model of FAD. A mouse model generated by intracerebroventricular (icv) administration of streptozocin (STZ), icv-STZ mouse, shows many aspects of SAD. Despite the wide use of these two models for AD research, differences in gene expression between them are not known. Here, we compared the expression of 84 AD-related genes in the hippocampus and the cerebral cortex between icv-STZ mice and 3xTg-AD mice using a custom-designed qPCR array. These genes are involved in APP processing, tau/cytoskeleton, synapse function, apoptosis and autophagy, AD-related protein kinases, glucose metabolism, insulin signaling, and mTOR pathway. We found altered expression of around 20 genes in both mouse models, which affected each of above categories. Many of these gene alterations were consistent with what was observed in AD brain previously. The expression of most of these altered genes was decreased or tended to be decreased in the hippocampus of both mouse models. Significant diversity in gene expression was found in the cerebral cortex between these two AD mouse models. More genes related to synaptic function were dysregulated in the 3xTg-AD mice, whereas more genes related to insulin signaling and glucose metabolism were down-regulated in the icv-STZ mice. The present study provides important fundamental knowledge of these two AD mouse models and will help guide future studies using these two mouse models for the development of AD drugs.

## Introduction

Alzheimer’s disease (AD) is the most common form of dementia, and the population of the affected people is growing due to increased life expectancy. The major pathological hallmarks of AD brain are senile plaques, consisting predominantly of extracellular amyloid-β (Aβ) peptides, and neurofibrillary tangles (NFTs), consisting of polymerized hyperphosphorylated tau protein. AD can be categorized into late-onset sporadic AD (SAD) and early-onset familial AD (FAD). FAD constitutes ∼1% of all AD cases and is caused by mutations in β-amyloid precursor protein (APP), presenilin 1 or 2 [1], [2]. Considerable progress has been made to unveil the pathogenesis and the molecular mechanisms of AD and to develop potential therapeutic approaches using different animal models. An extensively studied and used animal model is the triple transgenic mouse model, the 3xTg-AD mouse, which was generated by co-injecting two independent transgenic constructs encoding the Swedish mutations of human APP (APP_Swe_) and tau_P301L_ into single-cell embryos harvested from the mutant homozygous PS1_M146V_ knock in mice [3]. The 3xTg-AD mouse develops both amyloid plaques and NFTs in an age-and region-dependent manner [3]–[5].

SAD is a multifactorial disease caused by genetic, epigenetic, environmental and metabolic factors [6], among which impaired glucose metabolism and energy utilization are observed in the early stages of the disease [7], [8]. Accumulating studies suggest that brain insulin resistance exists in the brains of both AD and type 2 diabetes cases [9], [10] and, accordingly, AD has been proposed to be a brain-specific form of diabetes mellitus called “type 3 diabetes” [10], [11]. With the inspiration of the action of streptozocin (STZ) in the periphery, intracerebroventricular (icv) administration of STZ in rodents has been employed to induce brain insulin resistant state to generate an animal model of SAD [11]. Although some AD-related changes, such as learning and memory impairment and decreased glucose/energy metabolism, have been reported in the icv-STZ-rats/mice, more complete evaluation of this model is needed for its use for AD research and drug discovery.

**Table 1:** AD-related genes investigated in this study.

Category	Number of genes
APP processing and tau/cytoskeleton	18
Synapse	8
Apoptosis and autophagy	13
AD-related protein kinases	9
Glucose metabolism and O-GlcNAcylation	11
Insulin signaling	19
mTOR signaling	6
Total	84

AD is unique to humans. Although many AD mouse models have been reported, there is no single model that exactly mimics human AD. The 3xTg-AD mouse model has contributed greatly to our understanding of certain mechanistic pathways and pathologies of AD, especially the role of the mutations seen in FAD. However, this model falls short in studies of SAD, in which there are no mutations of any of these three genes. The icv-STZ model shows many aspects of abnormalities seen in SAD brain, but these animals are less studied and do not develop amyloid plaques or NFTs [12]–[15]. Thus, it is important to keep in mind the limitations when research data generated from using these AD animal models are interpreted.

**Figure 1:**
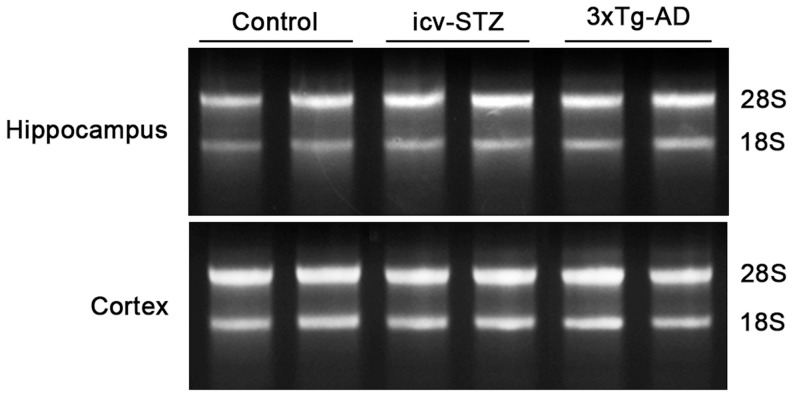
RNA integrity. Total RNA samples (1.5 µg/lane) extracted from mouse brains were separated in 1.2% native agarose gels and visualized under ultraviolet light.

It now becomes important to learn the similarities and differences between the commonly used 3xTg-AD mouse model representing FAD and the icv-STZ mouse model that mimics many aspects of SAD. To date, comparison between these two AD models has not been reported. In the present study, we compared mRNA expression profiles of 84 AD-related genes between the brains of the 3xTg-AD mice and the icv-STZ mice by using a custom-designed qPCR array.

**Figure 2:**
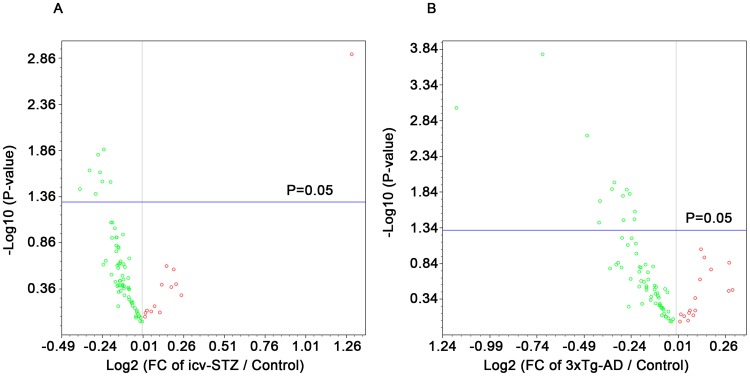
Volcano plots of the gene expression changes in icv-STZ mice and 3xTg-AD mice. Fold changes (FC) in gene expression of icv-STZ mice vs. control mice (A) and 3xTg mice vs. control mice (B). Each dot represents one gene. Green, down-regulated; red, up-regulated.

## Materials and Methods

### Animals and Study Outline

The breeding pairs of 3xTg-AD homozygous mice harboring PS1M_146V_, APP_Swe_ and tau_P301L_ transgenes and the wild type (WT) control mice (a hybrid 129/Sv × C57BL/6 mice) were obtained from Dr. F. M. LaFerla through the Jackson Laboratory (New Harbor, ME, USA) and were bred at the New York State Institute for Basic Research in Developmental Disabilities. Mice were housed (4∼5 animals per cage) with a 12/12 h light/dark cycle and with ad libitum access to food and water.

**Figure 3:**
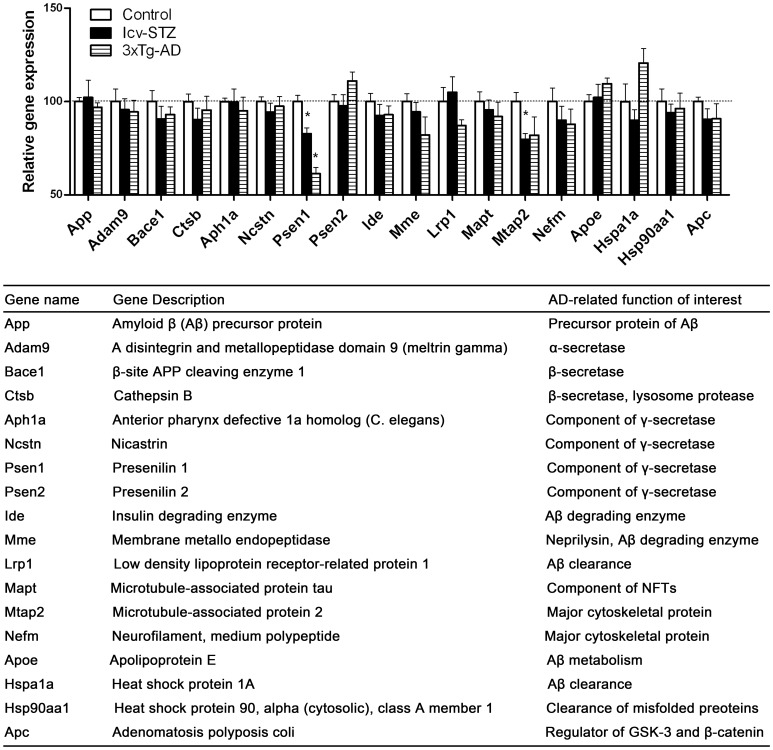
APP- and tau-related gene expressions.

The icv-STZ mice were produced by stereotaxic injection of STZ [2–deoxy–2–(3–(methyl–3–nitrosoureido)-D-glucopyranose), from Sigma-Aldrich (St. Louis, MO)] into the left lateral ventricle of the WT mice (female, 6 months old). Briefly, mice were first anesthetized using 2.5% avertin (2,2,2 tribromoethanol, Sigma-Aldrich) and then restrained onto a stereotaxic apparatus. The bregma coordinates used for injection were: −1.0 mm lateral, −0.3 mm posterior and −2.5 mm below. Each mouse received a single icv injection of STZ in 3.0 µl 0.9% saline into the left ventricle of the brain at a dose of 3.0 mg/kg. The mice were sacrificed 6 weeks after icv injection by decapitation, and the left cerebral cortices and hippocampi were immediately dissected and flash frozen in dry ice and then stored under −80°C until RNA extraction. The control WT mice and the 3xTg-AD mice (all female, 6 months old) were treated identically as above except they were injected with saline only.

**Figure 4:**
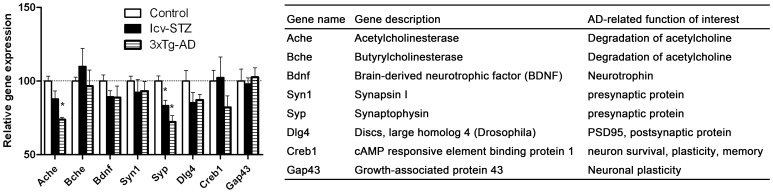
Synapse-related gene expressions in the hippocampus.

The animal experiments were approved by the Institutional Animal Care and Use Committee of the New York State Institute for Basic Research in Developmental Disabilities and were in accordance with the PHS Policy on Human Care and Use of Laboratory Animals (revised March 15, 2010).

### Total RNA Extraction

Total RNA was isolated from the cerebral cortical and hippocampal samples (four mice each group) using the RNeasy Mini kit (Qiagen, Valencia, CA) according to the manufacturer’s instructions. The RNA purity and integrity were determined by Nanodrop ND-1000 Spectrophotometer (Thermo scientific) and 1.2% agarose gel electrophoresis, respectively.

**Figure 5:**
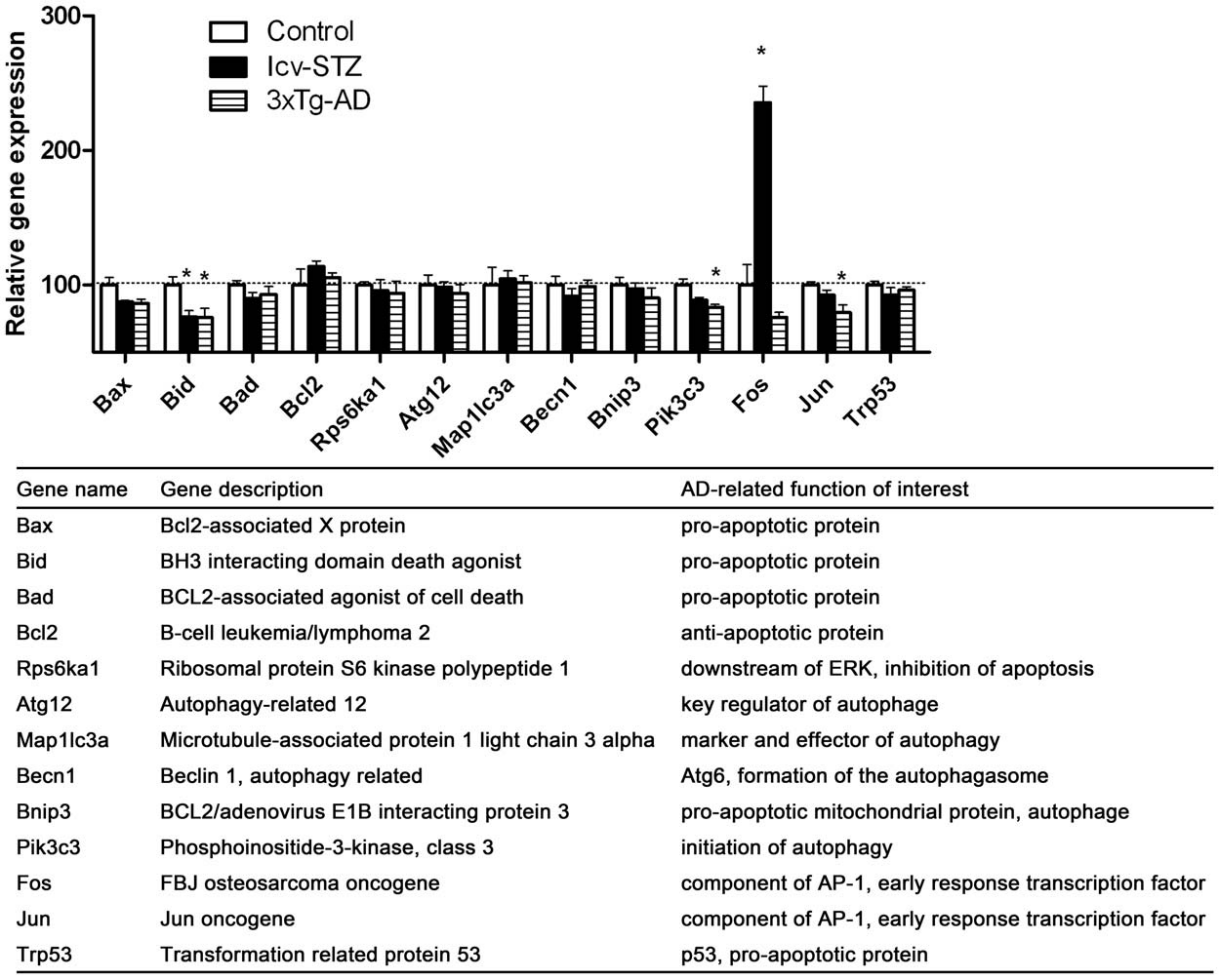
Expressions of apoptosis- and autophagy-related genes and transcription factors in the hippocampus.

### First Strand cDNA Synthesis and Quantitative Real-time PCR

Total RNA (1 µg) was subjected to reverse transcription reaction to synthesize cDNA using the RT^2^ First-Strand Kit (Qiagen) according to the manufacturer’s instructions. The diluted first-strand cDNA synthesis reaction mixture was used for real-time PCR using RT^2^ SYBR Green ROX qPCR Master Mix (Qiagen) and the custom-made 96-well PCR arrays in a Stratagene Mx3000p PCR detection system. Each array contains 84 genes that have been reported to be related to AD (Table 1), plus 5 housekeeping genes, 1 genomic DNA contamination control, 3 reverse transcription controls and 3 positive PCR controls, which allow for inter-well, intra-plate comparison.

**Figure 6:**
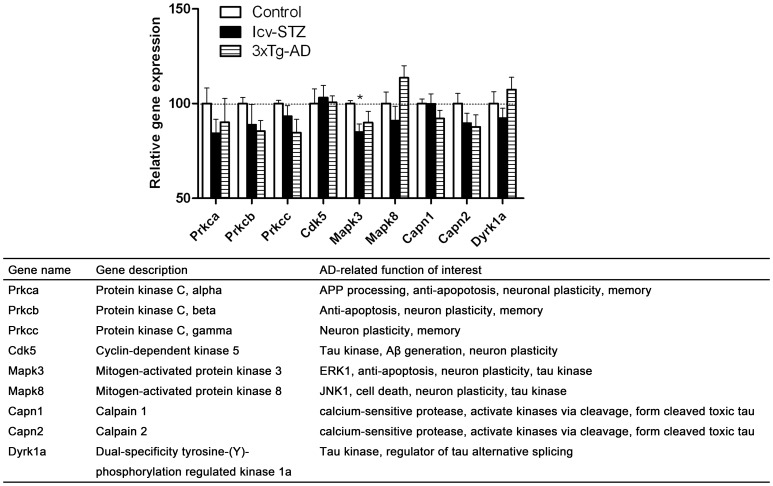
Gene expressions of AD-related protein kinases in the hippocampus.

### Statistic Analysis

The differences of gene expression were analyzed using Qiagen’s web-based PCR array data analysis system (http://pcrdataanalysis.sabiosciences.com/pcr/arrayanalysis.php), which is based on ΔΔC_t_ comparative method and unpaired two-tailed student *t* test. *P<0.05 was considered to be statistical significant.

**Figure 7:**
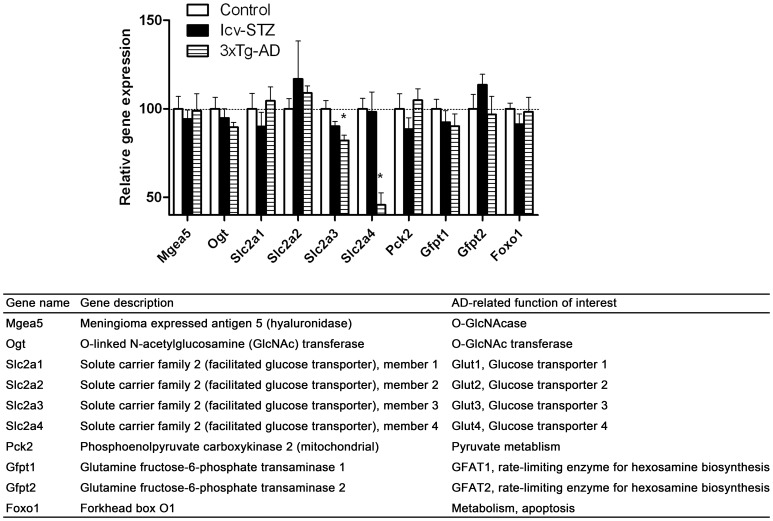
Glucose metabolism–related gene expressions in the hippocampus.

## Results

### Quality Control for RNA Extraction, Reverse Transcription and qRT-PCR

The quality of isolated RNA is critical for obtaining reliable results of qPCR arrays. We thus first evaluated the quality of RNA samples isolated from brain tissue by assessing the RNA purity and integrity. We found that the A260/A230 ratios were greater than 1.8, and the A260/280 ratios were greater than 1.9 of all the RNA samples we extracted (data not shown), indicating the acceptable RNA purities of our samples. Agarose gel electrophoresis showed sharp bands for 28S and 18S ribosomal RNA with the 28S/18S ratios of around 2 (Fig. 1), indicating the high quality of the RNA samples.

**Figure 8:**
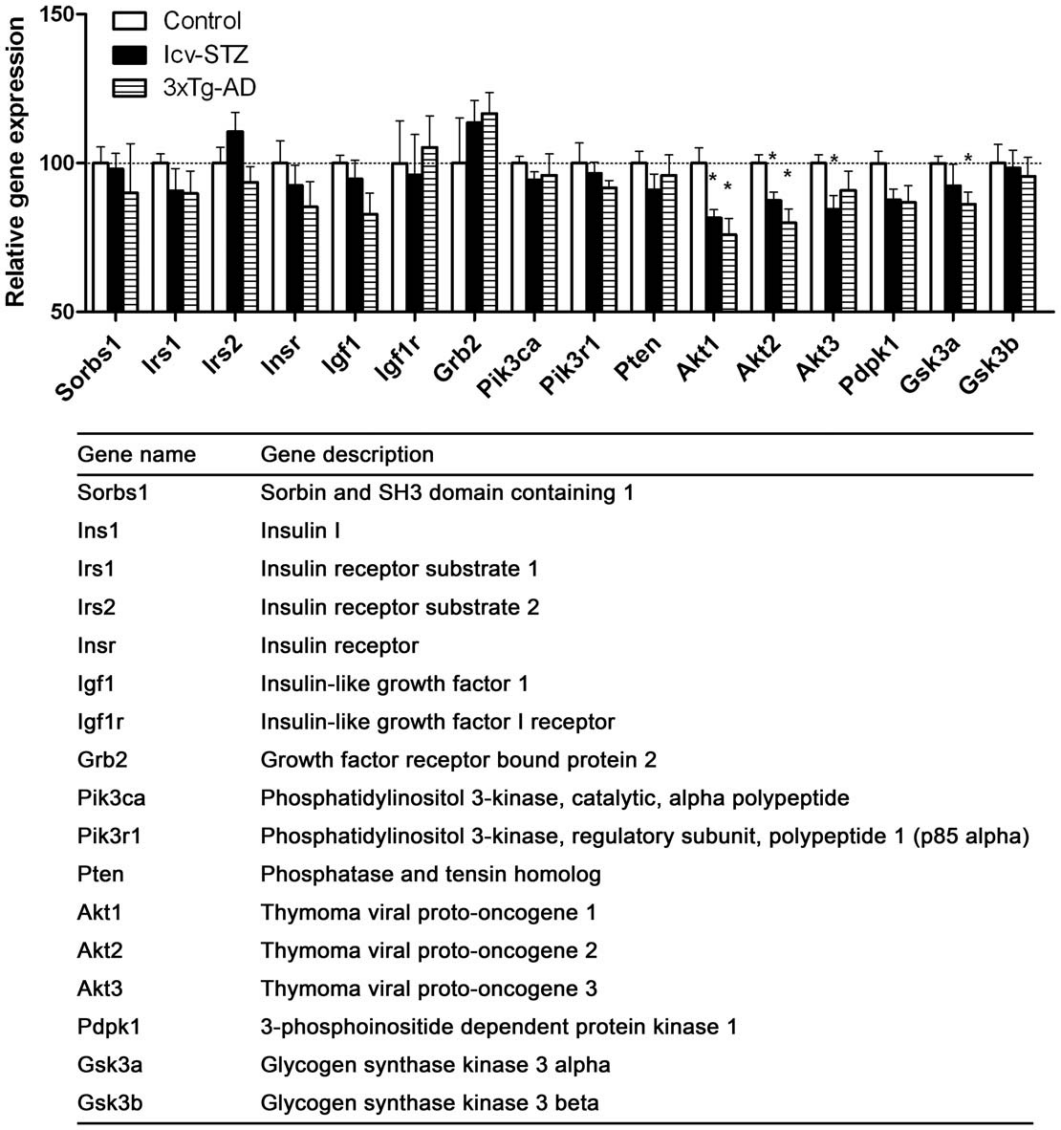
Insulin signaling–related gene expressions in the hippocampus.

Quality control of the PCR array was conducted according to manufacturer’s instructions, which tested the array’s reproducibility, RT efficiency and genomic DNA contamination. Every array we performed reached the expected criteria (data not shown).

### Overview of Gene Expression Profiles in the Hippocampus

The expression profile of 84 AD-related genes (Table 1) was analyzed by qPCR array of cDNA samples from the hippocampi of icv-STZ mice, 3xTg-AD mice and control mice. Among them, the data of four genes (*Igfap1, G6pc, Ins1, Chat*) were excluded from analyses because of the relative high threshold cycle (C_t_ >30) or no replication at all. We applied volcano plots for an overview of the gene expression changes in both groups when compared to control mice. As shown in Fig. 2, out of the 80 genes determined, 71 genes in the icv-STZ mice and 69 genes in the 3xTg-AD mice were down-regulated (green dots). The changes in the expression of 9 and 13 genes in the icv-STZ mice and the 3xTg-AD mice, respectively, reached statistical significance (above the horizontal lines). In order to have a detailed knowledge about these gene expression changes in the two AD models, these genes were further classified into seven subgroups: APP processing and tau pathology related genes, synapse function, apoptosis and autophagy, AD-related protein kinases, glucose metabolism, insulin signaling, and mTOR pathway.

**Figure 9:**
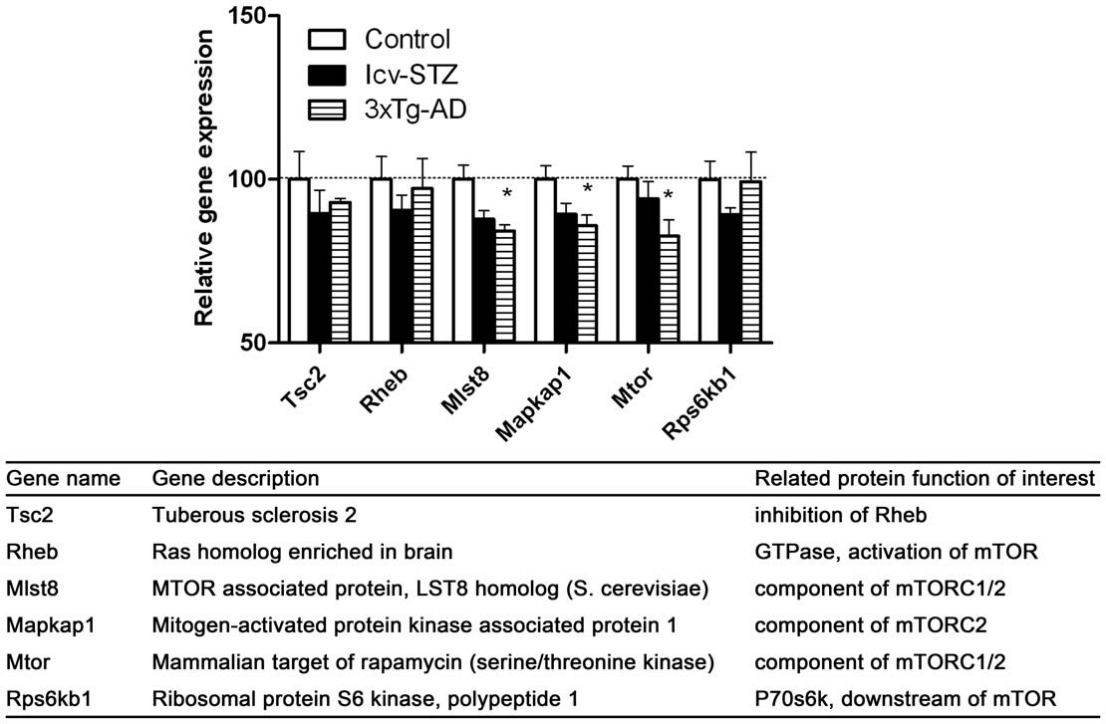
mTOR signaling–related gene expression in the hippocampus.

### APP- and Tau-related Gene Expressions in the Hippocampus

Abnormal processing of APP and abnormalities of tau and other cytoskeletal proteins are vital to the pathogenesis of AD. Thus, we first focused on expressions of those genes associated with the processing of APP, tau and cytoskeleton. We observed that, as compared with control mice, the expression of majority of the genes in this category appeared to be lower in both icv-STZ mice and 3xTg-AD mice (Fig. 3). Among them, the decreases in expression of *Psen1* and *Mtap2* were most obvious and reached statistical significance. It should be noted that the 3xTg-AD mice over-expressed mutated human presenilin 1, but the PCR array detected only mouse *Psen1*.

**Figure 10:**
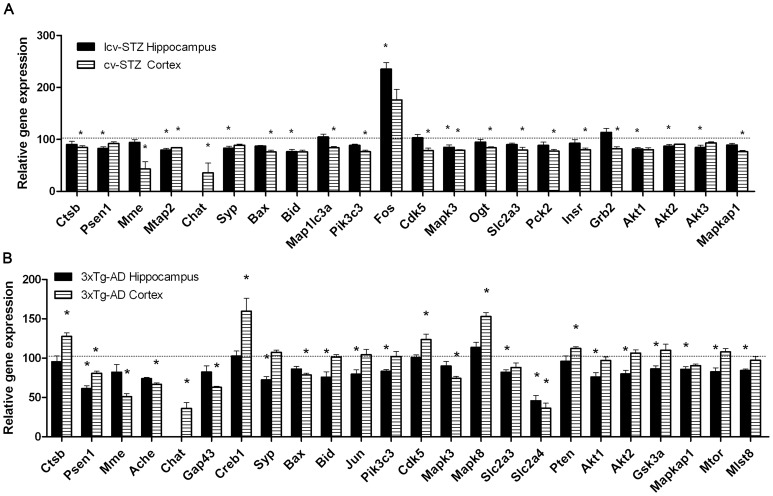
Comparison of altered AD-related gene expression profiles between the hippocampus and cerebral cortex. The relative levels of gene expression are shown, where those of the control mice are defined as 100. The hippocampal *Chat* data were excluded because of the high C_t_ values (30<Ct<37) that indicated unreliable data.

### Synapse-related Gene Expressions in the Hippocampus

Synaptic loss and dysfunction are believed to be the molecular basis of cognitive impairment in AD [16], [17]. Thus, we detected the expression of several synaptic markers, cholinesterase and BDNF. We found significantly decreased expression of synaptophysin (*Syp*) in the hippocampus of both icv-STZ and 3xTg-AD mice and of acetylcholinesterase (*Ache*) in 3xTg-AD mice (Fig. 4). Except for *Bche* and *Gap43*, the expression of other synapse-related genes studied was also reduced, but the reduction did not reach statistical significance. These results suggest synaptic dysfunction in the hippocampus of both icv-STZ and 3xTg-AD mice.

**Figure 11:**
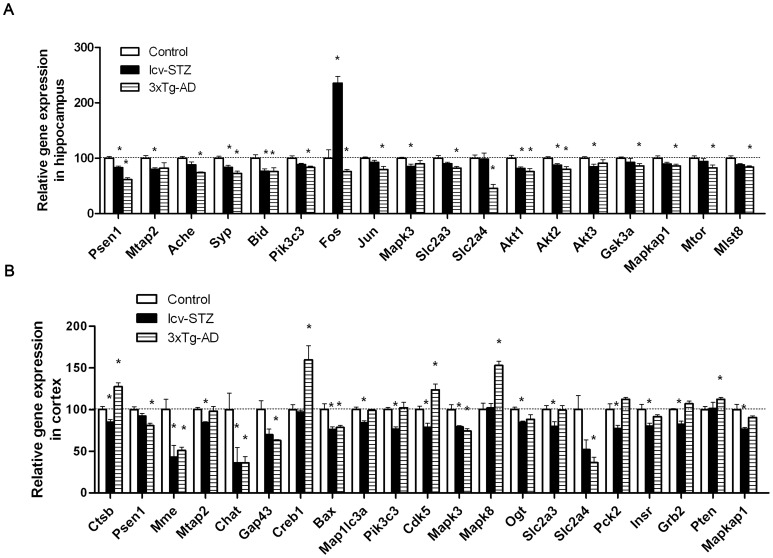
Comparison of altered AD-related gene expression profiles between the icv-STZ mice and the 3xTg-AD mice.

### Expressions of Apoptosis- and Autophagy-related Genes and Transcription Factors in the Hippocampus

Many studies have reported activation of apoptotic signaling in AD brain and suggest that apoptotic activity might be involved in neuronal loss [18]. Autophagy is an important process of removing intracellular aggregates through the lysosomal machinery, and its deregulation has been believed to contribute to the formation of hallmark AD pathologies [19]. Therefore, we studied the expression of apoptosis- and autophagy-related genes and transcription factors in the icv-STZ and the 3xTg-AD mouse brains. Among the pro-apoptotic genes studied here, we found a marked decrease of *Bid* expression in both icv-STZ and 3xTg-AD mice (Fig. 5). The expression of other pro-apoptotic genes was also decreased, but the decrease did not reach statistical significance. The expression of Pik3c3, which initiates autophagy, was also decreased in both both icv-STZ and 3xTg-AD mice, but the decrease only in 3xTg-AD mice reached statistical significance. Of the early response genes, the expression of *Fos* increased dramatically in the icv-STZ mice, but appeared to be decreased in the 3xTg-AD mice. Significant decrease of another early response gene, *Jun*, was also seen in the 3xTg-AD mice. We did not find significant changes in the expression of any autophagy-related genes in the hippocampus of either icv-STZ or 3xTg-AD mice.

### Gene Expressions of AD-related Protein Kinases in the Hippocampus

Most cell signaling transduction is switched on and off by phosphorylation and dephosphorylation of the signaling proteins. Protein phosphorylation also regulates neuronal plasticity, APP processing and tau aggregation [20]. Thus, we studied several protein kinases that have been demonstrated to be dysregulated in AD brain or to play some roles in the disease. We observed a trend of decrease in expression of protein kinase C isoforms, but only the decrease of ERK1 (*Mapk3*) in the icv-STZ mice reached the statistical significance (Fig. 6). We also studied calpains, calcium-activated brain proteases that are over-activated and consequently activate some protein kinases in AD brain [18], [21], [22], but no significant changes of their expression were seen in icv-STZ or 3xTg-AD mice.

### Glucose Metabolism–related Gene Expressions in the Hippocampus

Impairment of glucose/energy metabolism has been observed in the early stage of AD [7], [23], and the deterioration of cognitive deficits is associated with continuing decrease in glucose metabolism and spreading of the affected areas [23]. Therefore, we investigated the expression of several genes related to glucose metabolisms and O-GlcNAcylation. O-GlcNAcylation is an O-linked post-translational modification of nucleocytoplasmic proteins by a monosaccharide β-N-acetylglucosamine (O-GlcNAc) and is a sensor of intracellular glucose metabolism [24], [25]. Among the genes studied, we observed a marked reduction of the expression of *Slc2a3* and *Slc2a4,* which encode glucose transporter 3 and 4, respectively, in 3xTg-AD mice (Fig. 7). No significant alteration of these genes was seen in the icv-STZ mice. These results suggest reduced glucose uptake in the brains of the 3xTg-AD mice.

### Insulin Signaling–related Gene Expressions in the Hippocampus

Insulin plays an important role in energy metabolism and neuronal survival and plasticity in the brain [26], [27]. Impairment of brain insulin signaling has been observed in AD [9], [10], [26], [28], [29], which appears to contribute to neurodegeneration [28], [29]. We also found reduced protein level of some of the components of the insulin signaling in the cerebral cortex of icv-STZ rats [12]. Thus, we studied the expression of genes of the insulin signaling pathway. We found that the upstream components of the pathway (the first half listed in Fig. 8) were not significantly affected. However, the expression of the key downstream component, *Akt*, was found to be decreased in both the AD mouse models (Fig. 8). Reduced expressions of *Pdpk1* and *Gsk3a* were also observed, but only the reduction of *Gsk3a* in the 3xTg-AD mice reached statistical significance. These data suggest down-regulation of the basal insulin signaling in both the icv-STZ and the 3xTg-AD mice.

### mTOR Signaling–related Gene Expressions in the Hippocampus

mTOR signaling regulates a variety of neuronal functions and cross-talks with insulin signaling. Dysregulation of mTOR signaling has been implied in AD neurodegeneration [30], [31]. Here, we analyzed the main components of mTOR complex 1/2 (mTORC1/2), as well as their upstream factor *Tsc2* and downstream component P70S6k (*Rps6kb1*). We found decreased expression of mTORC1/2 components in both icv-STZ and 3xTg-AD mice, but only the decrease in the 3xTg-AD mice reached statistical significance (Fig. 9).

### Comparison of Altered Gene Expression Profiles between the Hippocampus and Cerebral Cortex

Besides the hippocampus, we also quantified AD-related gene expressions in the cerebral cortex using the same approach and compared the gene expression profiles between the hippocampus and the cerebral cortex. Fig. 10 shows all genes whose expressions were found to be altered in at least one of the two brain regions in the AD mouse models as compared to the control mice. We found that the expression of these genes were changed to the same direction in both brain regions, except the changes often did not reach statistical significance in one of the two brain regions. Overall, significant changes of more genes were seen in the cerebral cortex than the hippocampus of the icv-STZ mice (Fig. 10A), whereas significant changes of more genes were seen in the hippocampus than the cerebral cortex of the 3xTg-AD mice (Fig. 10B). These results suggest that, in respect to alterations of the AD-related gene expression, the brain regional abnormalities are different between the icv-STZ mice and the 3xTg-AD mice.

### Comparison of Altered Gene Expression Profiles between the icv-STZ Mice and the 3xTg-AD Mice

When we compared the overall changes of the AD-related gene expression, we found a clear similarity in down-regulation of many genes in the hippocampus between icv-STZ mice and 3xTg-AD mice. The expression of all these altered genes, except the early response gene *Fos*, was decreased or tended to be decreased in the hippocampus of both mouse models (Fig. 11A). The expression of *Fos* increased to more than twice in the icv-STZ mice, but it decreased in the 3xTg-AD mice.

More diversity was found in the cerebral cortex between the two AD mouse models. Among the 21 genes whose expressions were found to be altered in at least one of the two models, only four genes (*Mme, Chat, Mapk3, Bax*) showed decreased expression in both models (Fig. 11B). The expressions of the majority of these 21 genes were changed in the cerebral cortex of only one model, but not the other. There were two genes (*Ctsb* and *Cdk5*) whose expressions were decreased in the icv-STZ mice, but increased in the 3xTg-AD mice. More genes related to synaptic function were dysregulated in the cerebral cortex of the 3xTg-AD mice, whereas more genes related to insulin signaling (*Insr, Grb2, Pten*) and glucose metabolism (*Ogt, Slc2a3, Slc2a4, Pck2*) were down-regulated in the cerebral cortex of the icv-STZ mice.

## Discussion

Current understanding of the possible mechanisms of AD has led to many investigations of potential AD therapeutics. Mouse models are almost indispensable for the preclinical testing before any potential drugs go to clinical trials. In the present study, we used qPCR array and compared the alterations of gene expression profiles between icv-STZ mice, which show many aspects of SAD, and 3xTg-AD mice, the commonly used FAD mouse model. Our custom-designed qPCR array included 84 genes that have been implicated in AD. These genes are involved in APP processing and Aβ degradation, tau pathology, synaptic function, apoptosis and autophagy, AD-related protein kinases, glucose metabolism and O-GlcNAcylation, insulin signaling, and mTOR pathway.

A similar down-regulation of many genes in the brains of both the icv-STZ mice and the 3xTg-AD mice found in the present study suggests some common features of these two AD models, supporting the usefulness of both mouse models for AD research and drug development. However, the different alterations of several other genes, especially in the cerebral cortex, in these two models suggest diverse pathogenic pathways involved in SAD and FAD and also provide some useful information for the selection of these mouse models for AD drug development. By using microarray, down-regulation of genes related to cytoskeleton, synaptic plasticity, signal transduction, energy metabolism have been reported previously [32].

The deposition of Aβ results from an imbalance between its generation from APP and clearance. The most important Aβ degrading enzymes are insulin degrading enzyme (IDE) and neprilysin. A marked down-regulation of neprilysin expression in the brains of both mouse models observed in the present study may have contributed to the increased Aβ accumulation in the brain. In consistent to these observations, increased Aβ deposition in the wall of meningeal capillaries and cortical blood vessels was found in icv-STZ rats and icv-STZ Tg2576 mice [15], [33]. Increased aggregation of Aβ with the treatment of STZ was also seen in the hippocampus of Tg2576 mice [33]. The production of Aβ is regulated by APP expression and activities of α-secretase, β-secretse and γ-secretase. Among these genes, we found that the expression of presenilin 1 (*Psen1*), the catalytic subunit of γ-secretase, was down-regulated in the hippocampus of the icv-STZ mice and in both the hippocampus and the cerebral cortex of the 3xTg-AD mice. PS1 mutations are the cause of majority of the early onset familial AD [34]. Previous studies suggest that many PS1 mutations lead to the overproduction of Aβ_42_ because of its “gain-of-function” [35]. Recently, it is proposed that a “loss-of-function” mechanism may contribute to the synaptic dysfunction and neurodegeneration in AD [34], [36]–[38]. Mice after conditional knockout of brain PS1 show learning and memory impairment, synaptic dysfunction and neuronal death [37], [39]–[41], indicating that loss of essential functions of PS1 due to decreased PS1 expression or loss-of-function mutations of PS1 gene may be involved in the pathogenesis of AD. In the present study, the primers for all the genes in our qPCR array were mouse specific, so that only murine PS1 in 3xTg-AD mice was determined. While the mechanism of decreased PS1 expression in the icv-STZ mouse brains remains to be investigated, the down-regulation of murine PS1 expression in the 3xTg-AD mouse brains may represent a response to the over-expression of human PS1. It is interesting that cathepsin B, which has β-secretase activity [42], was found to be decreased in the icv-STZ mice, but increased in the 3xTg-AD mice. The up-regulation of cathepsin B in the 3xTg-AD mice may also be a response to the overexpression of APP.

Loss of synapses and dendritic spines correlates with cognitive decline in AD and precedes Aβ deposition, tangle formation and neuronal loss [16], [17]. We observed decreased expression of synaptic proteins, such as acetylcholineseterase (*Ache*), acetylcholine transferase (*Chat*), synaptophysin (*Syp*) and *Gap43*, in the brains of both the icv-STZ mice and the 3xTg-AD mice. The dysregulation of these synaptic proteins suggest impairment of synaptic function and is consistent with the cognitive impairment known in these mouse models [43]–[45]. The cholinergic pathway in the cerebral cortex and the basal forebrain are compromised in AD [45]. Marked reduction of *Chat* expression in the cerebral cortex of both the icv-STZ mice and the 3xTg-AD mice indicate a cholinergic impairment in these mice. The 3xTg-AD mice appeared to have more severe synaptic deficits in the cerebral cortex than the hippocampus, as *Gap43*, a component of the axon and presynaptic terminals, was also down-regulated in the cortex. Surprisingly, we observed increased expression of *Creb1*, which encodes the cAMP response element–binding protein (CREB), in the 3xTg-AD cerebral cortex. CREB is essential for maintaining synaptic plasticity and mediating the conversion of short-term memory to long-term memory [46]. Increased CREB expression does not necessarily lead to more CREB activity because the activity requires phosphorylation at Ser133 of CREB. The 3xTg-AD mice harbor a FAD PS1 (PS1_M146V_) knock-in. The increased *Creb1* expression we observed in the 3xTg-AD mice might have resulted from over-expression of the mutated PS-1, because this mutation affects CREB signaling [47].

Although activation of apoptotic signaling has been reported in AD brain [18], there is no evidence showing that the degenerative neurons die of apoptosis. It is generally believed that apoptosis signaling is activated as a response to AD pathogenic changes, but it fails to complete the pathway and kill neurons in AD brain. The icv-STZ mice and the 3xTg-AD mice show many alterations as what are seen in AD, but we found decreased, rather than increased, expression of pro-apoptotic genes, such as *Bid* and *Bax*. Autophagy is also dysregulated and is believed to contribute to the formation of hallmark AD pathologies [19], but we did not observe any significant changes of autophagy-related gene expressions. These observations demonstrate that neither of these two mouse models represents all brain abnormalities seen in AD.

Among those genes whose expressions were found to be increased in these two mouse models, *Fos* increased most remarkably in the icv-STZ mouse brains. *Fos* is an immediate early gene that represents a standing response to stimuli [48], [49]. The marked increase in *Fos* expression the icv-STZ mice might indicate the brain response to STZ injection, so that such an increase was not present in 3xTg-AD mice.

Impairment of glucose/energy metabolism has been observed in the early stage of AD, and it correlates with the severity of dementia [7], [23]. Gene expression analysis has showed reduced expression of genes that participate in the glucose energy metabolism in AD brain [50]. Proteomic approaches also found a significant downregulation of a set of proteins related to glucose/energy metabolism [51]. In the present study, we found marked decreases in expressions of *Slc2a3* and *Slc2a4* in the cerebral cortex in icv-STZ mice and in both the hippocampus and the cerebral cortex in 3xTg-AD mice. These two genes encode glucose transporters 3 and 4, respectively, which are critical for glucose uptake into the neuron. Thus, there could be a possible decrease of glucose uptake in the brains of these two AD mouse models. Decreased glucose transporters have been reported in AD brains [52], [53]. Of particular interest, glucose transporter 4 is regulated by insulin [54]–[57]. In rodent brain, insulin affects the use of glucose in specific regions of the brain through selective distribution of glucose transporter 4 [54]. It is possible that glucose transporter 4 also plays an important role in neuron glucose utilization in an insulin-dependent manner.

Recent studies have demonstrated that insulin not only is essential for regulating metabolism in the periphery, but also plays an important role in energy metabolism and neuronal survival and plasticity in the brain [26], [27]. Impairment of brain insulin signaling occurs in AD brain [9], [10], [26], [28], [29], [58] and appears to contribute to neurodegeneration [28]. We observed in this study that the majority of the genes involved in the insulin signaling pathway tended to be decreased in both the icv-STZ and the 3xTg-AD mice. The decrease of *Akt* expression, which encodes the key kinase of the insulin signaling pathway, was particularly remarkable and significant in the hippocampus. Downregulation of insulin signaling is expected in the icv-STZ mouse brains because STZ has been shown to induce insulin resistance in the periphery [59] and reduction of the insulin signaling pathway at the protein level in the brains of icv-STZ rats has been reported previously [12]. In the present study, we found that *Akt* expression was also decreased markedly in the hippocampus of 3xTg-AD mice. These findings suggest that a down-regulation of the basal insulin signaling may occur in both the icv-STZ and the 3xTg-AD mice.

mTOR is critical for long-lasting forms of synaptic plasticity and long-term memory [30]. We found down-regulation of several components of the mTOR complex in both icv-STZ and 3xTg-AD mice, although only the decrease in the 3xTg-AD mice reached statistical significance. These observations suggest deficient mTOR signaling in these mouse models. Down-regulation of mTOR signaling was also seen in the hippocampal slices of Tg2576 mice, which over-express the same mutated human APP as the 3xTg-AD mice, and this down-regulation correlates with impairment in synaptic plasticity [31]. Thus, the decrease in the expression of the mTOR complex in the 3xTg-AD mice might be related to over-expression of mutated human APP.

In conclusion, our observations demonstrate the similarities and differences between the icv-STZ and the 3xTg-AD mice, as well as provide detailed knowledge about the alterations of AD-related gene expression in these two mouse models that are commonly used for AD research. The present study provides important fundamental knowledge of these two AD mouse models and will help guide future studies using these two mouse models for the development of AD drugs.
